# Study of glycosylation of prostate-specific antigen secreted by cancer tissue-originated spheroids reveals new candidates for prostate cancer detection

**DOI:** 10.1038/s41598-020-59622-y

**Published:** 2020-02-17

**Authors:** Hiroko Ideo, Jumpei Kondo, Taisei Nomura, Norio Nonomura, Masahiro Inoue, Junko Amano

**Affiliations:** 1 Laboratory of glycobiology, The Noguchi Institute, Tokyo, 173-0033 Japan; 2 Department of Clinical Bio-resource Research and Development, Graduate School of Medicine, Kyoto University, Kyoto, 606-8501 Japan; 3 Department of Biochemistry, Osaka International Cancer Institute, Osaka, 541-8567 Japan; 4 Animal Models of Human Diseases, National Institutes of Biomedical Innovation, Health and Nutrition, Osaka, 567-0085 Japan; 5 Department of Urology, Osaka University Graduate School of Medicine, Osaka, 565-0871 Japan

**Keywords:** Prostate cancer, Glycobiology, Diagnostic markers

## Abstract

Prostate-specific antigen (PSA) is the most frequently used biomarker for the screening of prostate cancer. Understanding the structure of cancer-specific glycans can help us improve PSA assay. In the present study, we analysed the glycans of PSA obtained from culture medium containing cancer tissue-originated spheroids (CTOS) which have similar characteristics as that of the parent tumour to explore the new candidates for cancer-related glycoforms of PSA. The glycan profile of PSA from CTOS was determined by comparing with PSA from normal seminal plasma and cancer cell lines (LNCaP and 22Rv1) using lectin chromatography and mass spectrometry. PSA from CTOS was mostly sialylated and the content of *Wisteria floribunda* agglutinin reactive glycan (LacdiNAc) was similar to that of PSA derived from seminal plasma and 22Rv1. Conversely, concanavalin A (Con A)-unbound PSA was definitely detected from the three cancer origins but was almost negligible in seminal PSA. Two novel types of PSA were elucidated in the Con A-unbound fraction: one is a high molecular weight PSA with highly branched *N*-glycans, and the other is a low molecular weight PSA without *N*-glycans. Furthermore, the existence of Lewis X antigen group on PSA was indicated. These PSAs will be candidates for new cancer-related markers.

## Introduction

Prostate-specific antigen (PSA) is a glycoprotein that is exclusively produced by the prostate gland. PSA is normally found in semen, but men with prostate cancer often have a higher amount of PSA in the blood. PSA test is a screening test for prostate cancer; it measures the concentration of PSA in blood. The PSA levels also increase during non-cancerous conditions such as prostatitis (inflammation of the prostate), or benign prostatic hyperplasia (BPH, enlargement of the prostate). Men with PSA levels between 4 and 10 ng/ml have a 25% chance of developing prostate cancer, and this concentration range is known as the diagnostic grey zone. Therefore, a precise diagnosis that can distinguish cancer and inflammatory diseases is required.

Numerous studies have reported that aberrant glycosylation occurs in cancer cells rather than in normal cells^1,2^. These cancer-associated modifications of glycans are expected to help in a more precise diagnosis of cancer. Therefore, if aberrant glycans on PSA of cancer origin are present, they are attractive targets and several studies to determine them have been conducted^3^. Peracaula *et al*.^4^ analysed the glycans released from PSA in conditioned medium of LNCaP cancer cell line. The glycans were not sialylated, but they contained a Fucα1-2Gal residue and a high amount of GalNAcβ1-4GlcNAc (LacdiNAc). However, the glycans on PSA from the sera of prostate cancer patients were mostly sialylated and the glycan profiles are not the same as in the LNCaP cell^5,6^. It is well known that cell lines often acquire substantial bias and lose several characteristics of parental tumours during culture establishment and passage^7,8^; thus, the glycan profiles of such cells may have often changed from primary tumour.

To investigate whether glycan alterations of PSA occur in the patient’s sera, several studies have been performed using lectins that recognise α2,3-sialyl or LacdiNAc residues^9–12^. These studies revealed that the increase in these glycans was not necessarily significant in all patients. This is partly because some of these glycans are already present in normal cells. There is another issue in the case of testing serum samples; some PSA molecules come from non-cancerous tissue through a damaged barrier of tissue and blood vessel; particularly, many cases of prostate cancer accompany BPH. Therefore, it is hard to conclude that PSA in the patient’s blood is entirely caused by cancer. Furthermore, since the majority of PSA in blood forms a complex with α1-antichymotrypsin (ACT) which is glycosylated, the presence of PSA-ACT complex makes the measurement of the PSA glycans challenging.

In the present study, to overcome these issues, we analysed the PSA glycan structures from the culture medium containing cancer tissue-originated spheroids (CTOS) for the first time to the best of our knowledge. Using the CTOS method, highly purified and viable prostate cancer cells were efficiently prepared and cultured *in vitro*. CTOS formed xenograft tumours that retained the key features of the parental tumours^13^. Therefore, CTOS-derived PSA is considered to reflect glycan structures of the patient’s tumour. Furthermore, the obtained PSA should be produced only from cancer cells, completely free of non-cancerous cells. On comparison with the glycoforms of PSA from normal seminal plasma, CTOS, cancer cell lines LNCaP (lymph node metastasis) and 22Rv1 (localised to prostate), we can demonstrate the candidates of cancer-specific PSA glycoforms.

## Results

### Contents of sialylated and GalNAc-containing glycans of PSA from CTOS

**Figure 1:**
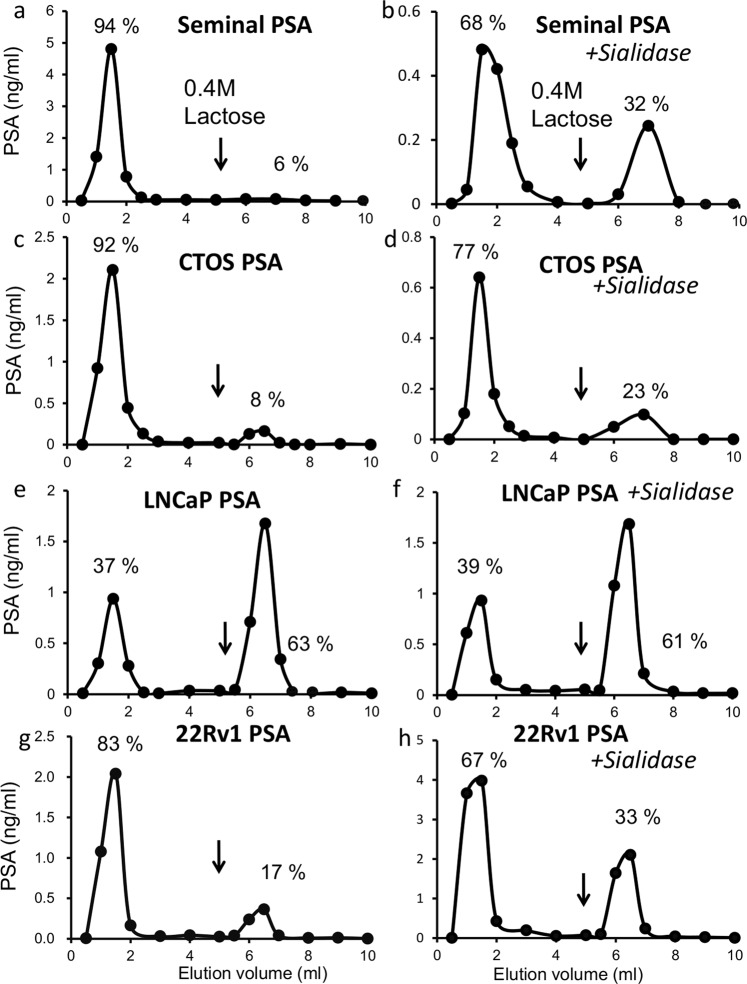
Elution profiles of PSA from cancer cells and seminal plasma before and after sialidase treatment on WFA column chromatography. (**a,b**) Seminal plasma. (c and d) CTOS. (**e,f**) LNCaP. (**g,h**) 22Rv1. Black arrows indicate the positions where the buffers were switched to those containing 0.4 M lactose.

To estimate the structures of glycan moieties in the secreted PSA from CTOS, we first applied the culture medium containing PSA to some types of lectin columns and measured the amount of PSA in the unbound (−) and bound (+) fractions, since the concentration of PSA in the CTOS culture medium was low (approximately 30 ng/ml) and the available volume was limited. PSA from seminal plasma of healthy men and LNCaP-conditioned medium were also simultaneously analysed using the same set of lectin columns. Several reports have shown that the LacdiNAc structure is abundantly expressed in LNCaP cancer cell and this was examined as the cancer-related glycomarker^14,15^; we measured the amount of PSA with LacdiNAc using *Wisteria floribunda* agglutinin (WFA) column chromatography. As shown in Fig. 1a,c, more than 90% of PSA from CTOS and seminal plasma passed through a WFA-Sepharose column and following *Arthrobacter* sialidase treatment, 23% and 32% of them bound to the column (Fig. 1b,d). On the contrary, more than 60% of PSA from LNCaP bound to the WFA column with or without sialidase treatment (Fig. 1e,f), indicating that more GalNAc residues in PSA from LNCaP exist than in PSA from CTOS and seminal plasma, and the residues in LNCaP are not sialylated. We have previously analysed seminal PSA using matrix-assisted laser desorption/ionisation-mass spectrometry **(**MALDI-MS) and found that 25% of PSA have one LacdiNAc in its *N*-glycan moiety^16^. The WFA column chromatography shows a good agreement with MALDI-MS analysis. The result of WFA column showed that the content of GalNAc in PSA from CTOS was not high unlike PSA from LNCaP. Since the behaviour of PSA from LNCaP on a WFA column differs from that of PSA from semen and CTOS, we examined another cancer cell line, 22Rv1. More than 80% of PSA from 22Rv1 passed through a WFA-Sepharose column (Fig. 1g) and 33% bound to the column after *Arthrobacter* sialidase treatment (Fig. 1h). These results show that in these cancer cells, other than LNCaP, LacdiNAc residue was not more than normal cells.

### Concanavalin A (Con A)-unbound glycoforms in PSA from cancer cells

**Figure 2:**
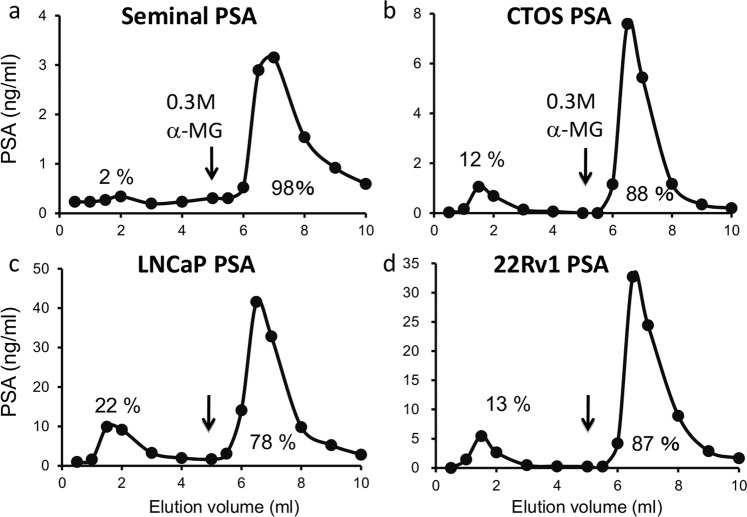
Elution profiles of PSA from cancer cells and seminal plasma on Con A column chromatography. (**a**) Seminal plasma. (**b**) CTOS. (**c**) LNCaP. (**d**) 22Rv1. Black arrows indicate the positions where the buffers were switched to those containing 0.3 M α-MG.

We applied PSA from CTOS and other sources to a Con A column. Consequently, 12% of PSA from CTOS, 13% of PSA from 22Rv1 and 22% of PSA from LNCaP passed through a Con A column (Con A (−) fraction), while less than 2% of seminal PSA passed (Fig. 2). Seminal PSA derived from different lots and companies including from WHO International Standard was also applied to a Con A column, and the Con A (−) fraction of PSA did not exceed 2% (data not shown). From these results, a significant amount of Con A-unbound PSA secretion was commonly identified in cancer cells.

### High and low molecular weight forms of PSA in Con A (−) fraction of cancer cells

**Figure 3:**
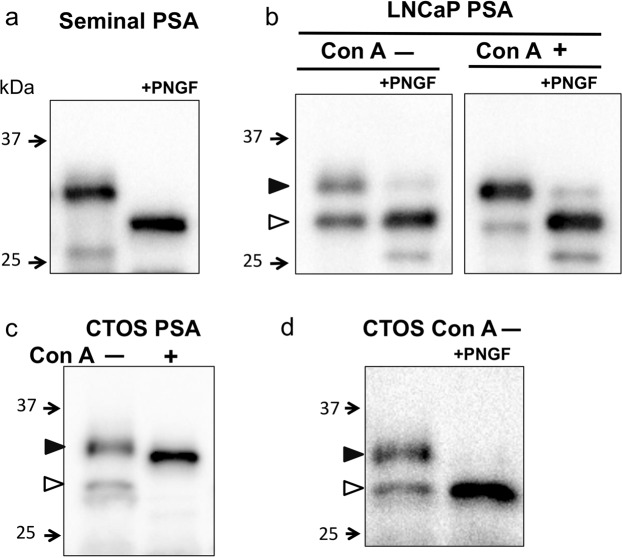
Western blot analysis of PSA from seminal plasma and cancer cells. (**a**) Seminal PSA with and without PNGF treatment. (**b**) PSA from LNCaP in Con A (−) and (+) fraction with and without PNGF treatment. (**c**) Immunoprecipitated PSA in Con A (−) and (+ fraction from CTOS. (**d**) Immunoprecipitated PSA in Con A (−) fraction from CTOS with and without PNGF treatment. The position of each high molecular and low molecular PSA was indicated by closed and open triangle, respectively. Full-length blots are presented in Supplementary Fig. S4.

Next, we analysed PSA molecules in Con A (−) and (+) fractions by Western blotting. Since seminal PSA contained almost no Con A (−) fraction, we analysed it without Con A chromatography. The seminal PSA had the molecular mass of 31 kDa, and the molecular mass changed to 29 kDa after PNGase F (PNGF) treatment (Fig. 3a). The PSA from LNCaP in Con A (−) fraction separated into the molecular masses of 32 kDa (Fig. 3b, closed triangle) and 29 kDa (Fig. 3b, open triangle), while Con A (+) fraction had molecular mass of 31 kDa (Fig. 3b) that is the same as seminal PSA. Following PNGF treatment, both high molecular weight forms (32 and 31 kDa) changed to the low molecular weight form (29 kDa). On the other hand, the majority of 29 kDa (open triangle) in Con A (−) fraction did not change, suggesting that it was either with shortened or without *N*-glycans (Fig. 3b).

**Figure 4:**
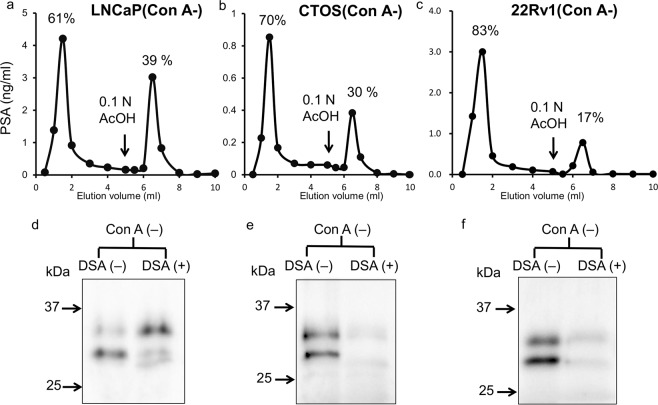
Analysis of PSA in Con A (−) fraction from cancer cells. Elution profiles of PSA in Con A (−) fraction from cancer cells on DSA column (upper panel). (**a**) LNCaP. (**b**) CTOS. (**c**) 22Rv1. PSA from cancer cells were treated with sialidase before Con A column chromatography. Black arrows indicate the positions where the buffers were switched to 0.1 M acetic acid containing 0.1 mg/ml BSA. Western blot analysis of PSA in DSA (−) and (+) fractions (lower panel). (**d**) LNCaP. (**e**) CTOS. (**f**) 22Rv1. The (−) and (+) fractions of DSA column of CTOS (**e**) and 22Rv1 (**f**) were concentrated and were immunoprecipitated and eluted with SDS sample buffer as described in “Methods”. Full-length blots are presented in Supplementary Fig. S4.

The PSA from CTOS also had the molecular mass of 32 kDa (Fig. 3c, closed triangle) and 29 kDa (Fig. 3c, open triangle) in Con A (−) fraction and 31 kDa in Con A (+) fraction (Fig. 3c). The low molecular weight form (29 kDa) in Con A (−) fraction migrated at the same position as the PNGF treated seminal PSA. Following PNGF treatment, the high molecular weight form (32 kDa) changed to the low molecular weight form (29 kDa) (Fig. 3d). As for PSA from 22Rv1, the Con A (−) fraction also contained the molecular masses of 32 kDa and 29 kDa (Fig. 4f).

The two forms of PSA with different molecular masses were found in Con A (−) fractions in CTOS, 22Rv1 and LNCaP cancer cells. Any high molecular PSA in Con A (−) fractions are slightly larger than the ones in the Con A (+) fractions. The low molecular PSA is considered to be either with shortly *N*-glycosylated or without *N*-glycans.

### Multi-antennary and multi-fucosylated *N*-glycans in PSA from LNCaP cancer cell

**Figure 5:**
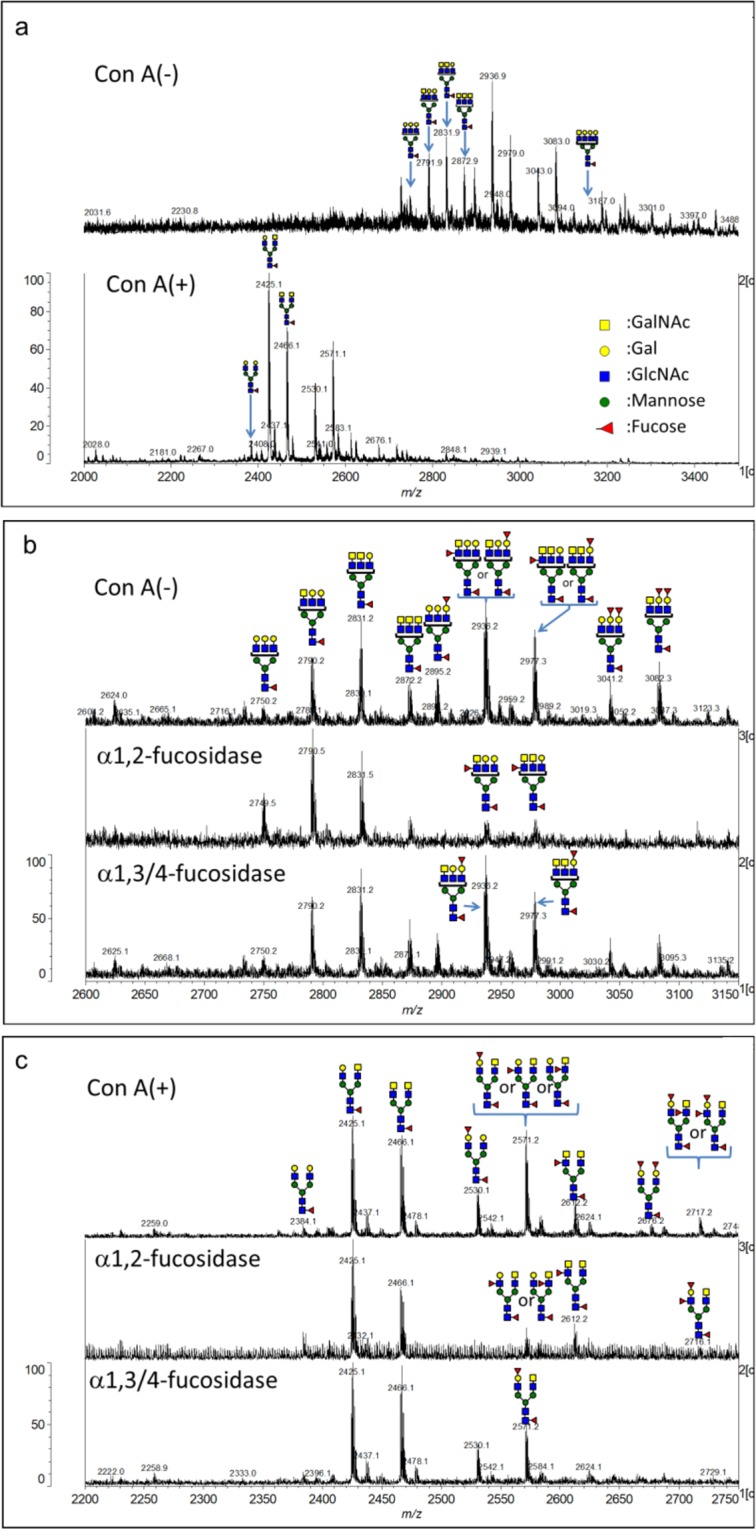
Analysis of PSA glycopeptides from LNCaP using MALDI-MS. (**a**) MALDI-TOF MS spectra of glycopeptides in Con A (−) (upper) and Con A (+) (lower) fractions. (**b**) Enlarged spectra (*m/z* 2600 to 3150) of glycopeptides in Con A (−) fraction without (upper), with α1,2-fucosidase(middle) and with α1,3/4-fucosidase(lower). (**c**) Enlarged spectra (*m/z* 2200 to 2750) of glycopeptides in Con A (+) fraction without (upper), with α1,2-fucosidase (middle), and α1,3/4-fucosidase (lower). Mass spectra were acquired in negative ion mode.

We studied the *N*-glycan structures from LNCaP cell in detail. Since the amount of PSA in CTOS medium is limited, we immunoprecipitated PSA of the Con A (−) and (+) fractions from a large amount of conditioned medium of LNCaP cell and analysed them using MALDI-MS. For profiling the glycans at the *N*-glycosylation site of PSA, the peptide IRNKS (positions 43–47) with a glycan was obtained by thermolysin digestion. Glycan structures were confirmed using MALDI-MS^n^. A glycopeptide profile spectrum is shown in Fig. 5a. PSA glycopeptides of the Con A (+) fraction comprised bi-antennary glycans containing 0–2 LacdiNAc residues and had 0–2 fucose residues in the outer ends in addition to the core fucose. The glycopeptides in the Con A (−) fraction was confirmed to have no bi-antennary glycans. Instead, glycans with three or more branches were detected. The glycopeptides with tri-antennary glycans containing 0–3 LacdiNAc residues had 0–3 fucose residues in the outer ends in addition to core fucose. Together with the results of lectin chromatography, glycans with GalNAc in PSA from LNCaP were shown to be more abundant than those in normal PSA.

For determining the linkages of fucoses in the Con A (−) fraction, we treated glycopeptides with α1,2- or α1,3/4-specific fucosidases. Ions at *m/z* 2895, 3041 and 3082 disappeared after α1,2-fucosidase (Fig. 5b, middle) but not α1,3/4-fucosidase (Fig. 5b, lower) digestion suggesting the α1,2-fucosylation. Ions at *m/z* 2936 and 2977 changed by both fucosidases digestion suggesting the existence of both α1,2- and α1,3(4)-linked fucoses (Fig. 5b, middle and lower). We also analysed the linkage of fucoses of glycopeptides in the Con A (+) fraction. The ion at *m/z* 2612 was digested by α1,3/4-fucosidase (Fig. 5c, lower) but not by α1,2-fucosidase (Fig. 5c, middle) suggesting the existence of α1,3(4)-linked fucose. Also, ions at *m/z* 2530 and 2676 should have α1,2-linked fucose, and fucoses of ions at *m/z* 2571 and 2717 should be α1,2- and α1,3(4)-linked. These glycopeptides are considered to come from high molecular PSA.

### Non-*N*-glycosylated form of PSA in the Con A (−) fraction from LNCaP cancer cell

We further analysed the low molecular PSA in the Con A (−) fraction using MALDI-MS. If an endo-β-*N*-acetylglucosaminidase is present, it should have cleaved between the two *N*-acetylglucosamine residues in the *N*-linked diacetyl chitobiose core of the glycan, generating a truncated sugar molecule with one *N*-acetylglucosamine (±fucose) on the Asn. In another case, a peptide : *N*-glycanase (PNGase) that cleaves the amide bond between the innermost GlcNAc and the Asn residue, should have generated a de-*N*-glycosylated protein, resulting in conversion of Asn to Asp. We digested PSA in the Con A (−) fraction from LNCaP with Asp-N, which specifically cleaves the peptide bonds at the amino side of aspartic acid or cysteic acid residues, and analysed the peptides using MALDI-MS. If Asn was converted to Asp by PNGase, we should detect the peptide peak (*m*/*z* 1847.8) that corresponds with the peptide 45–60; however, we could not actually detect this peptide peak (data not shown). We prepared cDNA and analysed the sequence of PSA from CTOS and LNCaP. No mutations were found at the *N*-glycosylation potential site (data not shown).

**Figure 6:**
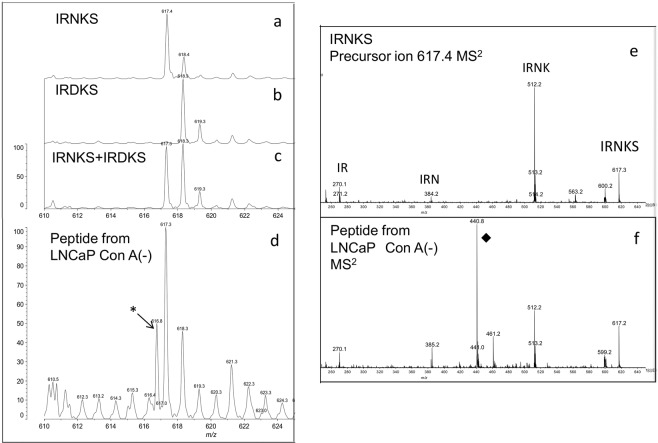
MALDI-MS spectra of PSA peptides. (**a**) IRNKS. (**b**) IRDKS. (**c**) Mixture of IRNKS and IRDKS. (**d**) PSA peptides from LNCaP in Con A (−) fraction. (**e**) MS/MS spectrum of IRNKS. (**f**) MS/MS spectrum of PSA peptides from LNCaP in Con A (−) fraction of precursor ion at *m/z* 617.4 corresponding to the peptide IRNKS. The peak (♦) comes from contaminating peptide that is detected of precursor ion at *m/z* 616.8 corresponding to the peptide of the peak (*) in (**d**). Mass spectra were acquired in positive ion mode.

Since synthetic peptides IRNKS and IRDKS were retained on GL-Tip-Amide together with glycopeptides because of their hydrophilic character, we inspected if truncated glycopeptides or non-glycosylated peptides 43–47 exist on the spectra of thermolysin-digested PSA from LNCaP. The synthesised peptide IRNKS have isotopic mass peaks (*m*/*z* 617.4 and 618.4) (Fig. 6a), and the synthesised peptide IRDKS have isotopic mass peaks (*m*/*z* 618.3 and 619.3) (Fig. 6b). Since the ion at *m/z* 618 comes from both peptides, it cannot be clearly distinguished between only IRNKS and the mixture of IRNKS and IRDKS (Fig. 6c). In the digested PSA from LNCaP, we detected the ions at *m*/*z* 617.3 and 618.3 (Fig. 6d) but not the peaks corresponding with glycopeptides GlcNAc-IRNKS (*m*/*z* 820.4) or Fuc-GlcNAc-IRNKS (*m*/*z* 966.5) (data not shown). The MS^2^ spectrum of the precursor ion (*m*/*z* 617) in Fig. 6d shows the same fragments as the peptide IRNKS (Fig. 6e,f). However, we could not determine whether it was only IRNKS or the mixture of IRNKS and IRDKS as already mentioned. Therefore, we analysed them using liquid chromatography-mass spectrometry (LC-MS) because it was expected that IRNKS and IRDKS are eluted at different positions from the LC column.

### Identification of non-glycosylated PSA peptide from LNCaP as IRNKS

**Figure 7:**
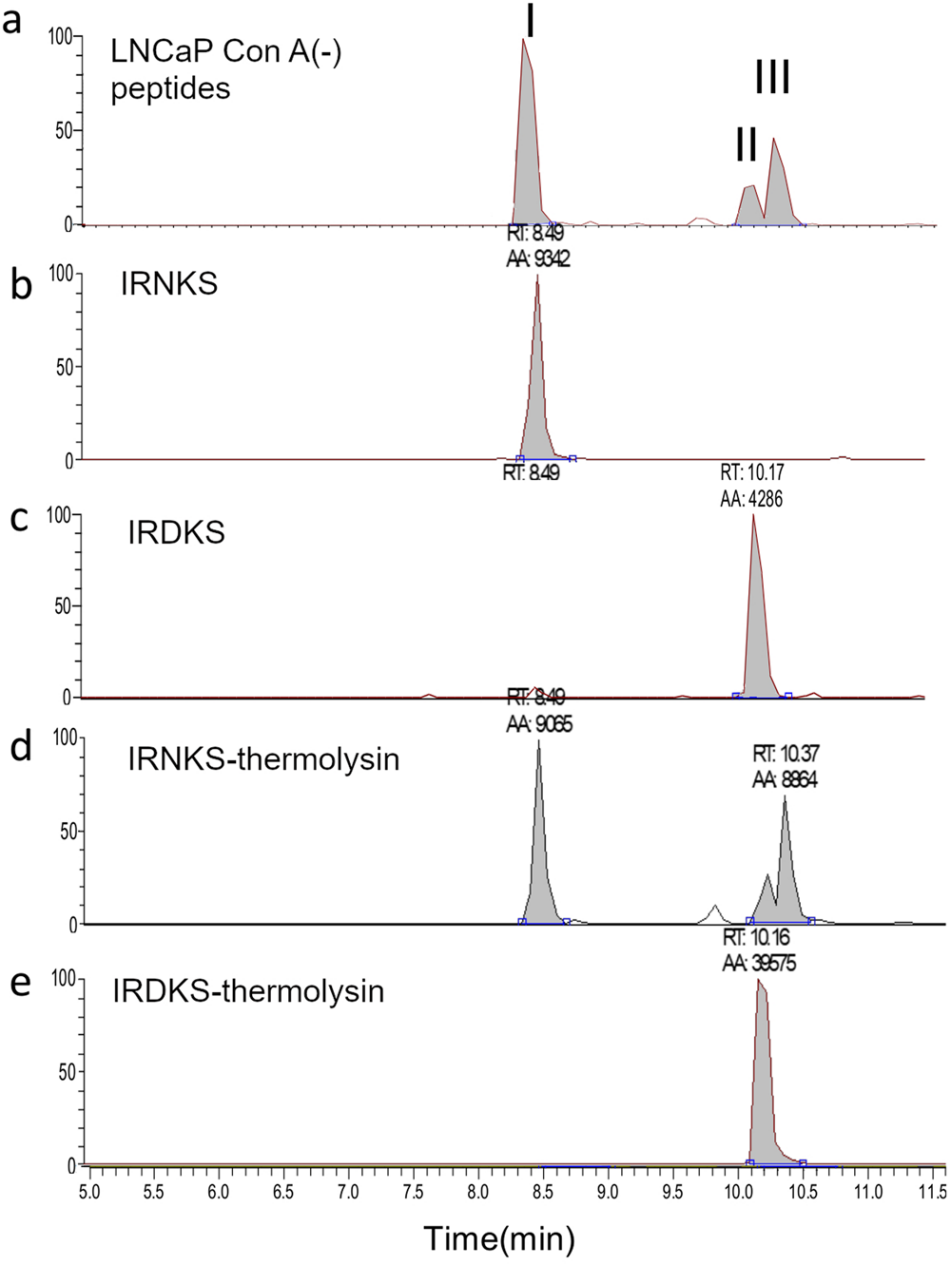
LC-MS chromatograms of synthetic peptides and PSA peptides. (**a**) PSA peptides in Con A (−) fraction of LNCaP. (**b**) IRNKS. (**c**) IRDKS. (**d,e**) Synthetic peptides were treated with thermolysin and purified in the same manner as peptides of PSA from LNCaP.

The peptides digested by thermolysin of the Con A (−) fraction of PSA from LNCaP were applied to LC-MS analysis and a peak (I) at the same retention time as that with standard IRNKS was detected (Fig. 7a,b). A peak (II) at the same retention time as that with standard IRDKS was also detected (Fig. 7a,c). The synthetic peptide IRNKS eluted as a single peak at 8.49 min (Fig. 7b), however, after thermolysin treatment IRNKS contained other two peaks at 10.17 and 10.37 min (Fig. 7d). On the contrary, the synthetic peptide IRDKS eluted at the same single position before and after thermolysin treatment (Fig. 7c,e). Therefore, the twin peaks II and III in Fig. 7a from the LNCaP Con A (−) peptides should be partly produced from IRNKS during thermolysin digestion, suggesting that conversion of Asn to Asp and iso-Asp^17^.

### Presence of high molecular PSA glycans that do not bound *Datura stramonium* agglutinin (DSA)

To further examines the *N*-glycan structures of the high molecular PSA in Con A (−) fraction from cancer cells, we applied them to a DSA column since DSA recognises the multi-antennary *N*-glycans that Con A does not recognize^18^. Majority of the high molecular PSA in the Con A (−) fraction from LNCaP bound to a DSA column suggesting that they have multi-antennary *N*-glycans (Fig. 4a,d). In the case of PSA from CTOS and 22Rv1, significant amounts of the high molecular form of the Con A (−) fraction in addition to non-*N*-glycosylated PSA were detected in DSA (−) fraction (Fig. 4e,f). The tri-antennary glycans with Galβ1-4GlcNAcβ1-4(Galβ1-4GlcNAcβ1-2)Manα1-3(2,4 branched) and Galβ1-4GlcNAcβ1-6(Galβ1-4GlcNAcβ1-2)Manα1-6(2,6 branched) interact with DSA^18^. In fact, we confirmed that both tri-antennary pyridylamino-sugars retained the DSA column (data not shown). Reportedly, DSA reduces its affinity by fucosylation on GlcNAc (Galβ1-4(Fucα1-3)GlcNAc: Lewis X (Le^X^) structure) attached to tri- and tetra-antennary glycans^19^. PSA from LNCaP contains not only α1,2-fucosylated but also α1,3-fucosylated glycans as mentioned in above section and there is possibility of higher α1,3-fucosylation to outer sugar chains of CTOS and 22Rv1 than LNCaP.

### Le^X^ structure expression in PSA from CTOS and 22Rv1

Since the possibility of α1,3-fucosylation of GlcNAc was suggested from the result of the DSA chromatography of PSA from CTOS and 22Rv1, we examined the α1,3-fucosylation of bi-antennary glycans of Con A (+) fractions, which account for more than 80% of total PSA. Galectin-1 recognises the non-reducing terminal galactoses of bi- or multi-antennary glycans; however, its affinity reduced by α2,6-sialylation of the terminal galactose and/or α1,3(4)-fucosylation of the GlcNAc adjacent to galactose^20,21^. Therefore, we examined the effect of sialidase and α1,3/4-fucosidase on the galectin-1 binding patterns.

**Figure 8:**
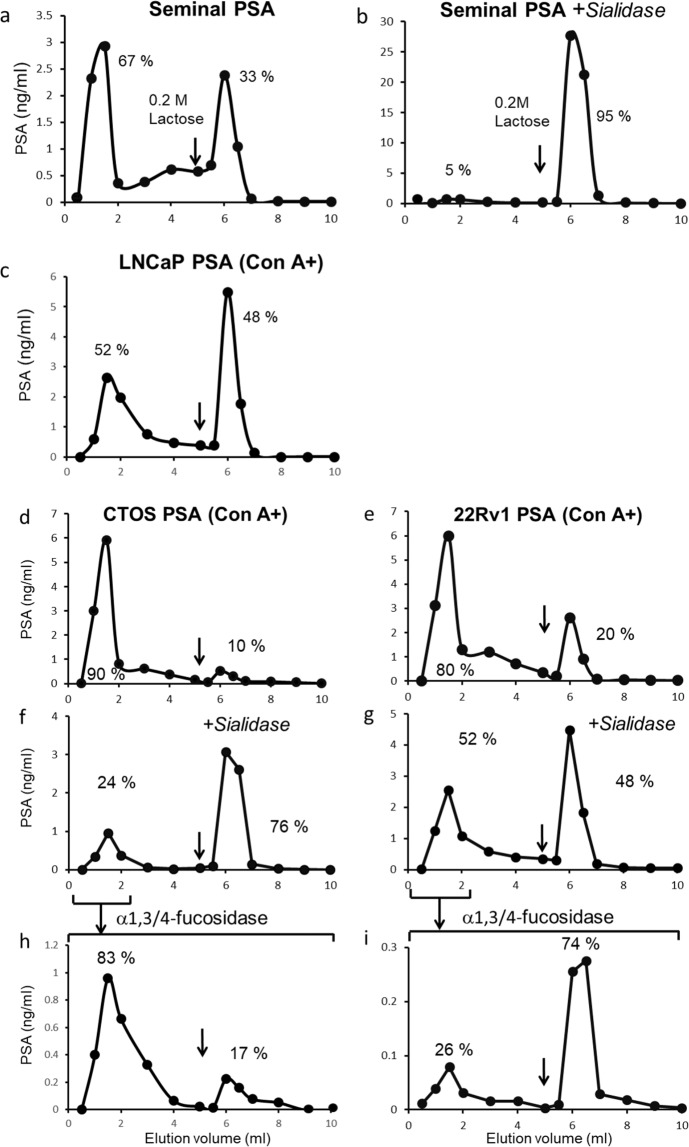
Elution profiles of PSA on galectin-1 column chromatography. (**a**) Seminal PSA. (**b**) sialidase-treated seminal PSA. (**c**) LNCaP, (**d**) CTOS and (**e**) 22Rv1 PSA in Con A (+) fraction. (**f,g**) sialidase-treated PSA from CTOS and 22Rv1 in Con A (+) fraction. (**h,i**) α1,3/4-fucosidase treated PSA of galectin-1 (−) fractions of sialidase-treated PSA from CTOS(f) and 22Rv1 (**g**). Black arrows indicate the positions where the buffers were switched to switched to those containing 0.2 M lactose.

When we applied seminal PSA to a galectin-1 column, only 30% of intact PSA but almost all of sialidase-treated PSA bound to a galectin-1 suggesting α2,6-sialylated galactose is the major form but no α1,3-fucosylation (Fig. 8a). In the case of the Con A (+) fraction of PSA from LNCaP (Fig. 8c), about half of it did not bind to a galectin-1 column even no sialylation. This agrees with the results from MS analysis (Fig. 5) that GalNAc-terminated and α1,3(4)-fucosylated GlcNAc adjacent to galactose glycans which are passed through a galectin-1 column were found. The majority of PSA from 22Rv1 and CTOS did not bind to a galectin-1 column (Fig. 8d,e) and some of them did not bind even after sialidase treatment (Fig. 8f,g). However, after α1,3/4-fucosidase digestion of the galectin-1 (−) fraction of sialidase-treated PSA, some of them bound to the galectin-1(Fig. 8h,i), suggesting an existence of α1,3(4)-fucosylation of GlcNAc.

Since higher levels of α1,3-fucosyltransferases (FT) 3, 6 and 7 were reported in prostate cancer cells^22,23^, we also examined the expression of α1,3-FT3s using real-time PCR. Consequently, CTOS and 22Rv1 express more α1,3-FTs than that of LNCaP cells (Supplementary Fig. S1) supporting the elevation of α1,3(4)-fucosylation in CTOS and 22Rv1.

It has been reported that PSA has only one *N*-glycosylated site even it has three potential sites for O-glycosylation^24^. However, there are studies which suggested the presence of core 1 *O*-glycan^25^ and core 2 *O*-glycan with sLe^X^^26^. Therefore, we examined for the presence or absence of *O*-glycan on PSA because LNCaP cell can produce core 2 *O*-glycan with polylactosamine which is recognized by galectin-1^27^. To compare the molecular mass with the PNGF treated PSA from LNCaP, we prepared recombinant non-glycosylated PSA in *E. coli*. The molecular mass of the recombinant pro-form of PSA was 26971 calculated from the amino acid sequence for the molecule, however, it was shown as 29.2 kDa by SDS-PAGE suggesting an overestimation of 2.2 kDa occurs (Supplementary Fig. S2). Based on this, the real molecular mass of PNGF treated PSA from LNCaP is estimated to be 26.6 kDa because it was determined as 28.8 kDa by SDS-PAGE (Supplementary Fig. S2). From the amino acid sequences the molecular masses of pro-form and active form of PSA are calculated as 26840 and 26089, respectively. Since some of the secreted pro-form of PSA turned to the active form^28^, average molecular mass of peptide portion is estimated to be around 26.5 kDa. The finding that this molecular mass of peptide alone was in good agreement with the mass of the PNGF treated PSA from LNCaP concluded that the presence of core 2 *O*-glycan with polylactosamine (glycan mass is calculated as more than 1.4 kDa) on PSA from LNCaP is negligible.

## Discussion

By analysing the *N*-glycan structures of PSA from CTOS, we found the new candidates of cancer specific PSA with highly branched *N*-glycans and also with no *N*-glycans. PSA from CTOS was mostly sialylated and the content of WFA reactive *N*-glycans (LacdiNAc) is similar to that of PSA derived from normal seminal plasma and 22Rv1 (Fig. 1), suggesting that an increase in LacdiNAc and a decrease in sialylation are not common in all prostate cancers. However, PSA molecules that could not interact with Con A were secreted from both CTOS and other cancer cells, which is almost negligible in seminal PSA from healthy men (Fig. 2).

Some studies have detected non-glycosylated PSA using 2D-electrophoresis; however, they did not directly detect non-*N*-glycosylated peptide from the PSA^29,30^. Therefore, the possibility of shortly glycosylated forms of PSA cannot be denied, and no further studies were conducted. In previous studies, amounts of PSA in both Con A (−) and (+) fractions were measured comparing between lysates from tissues of prostate cancer and BPH^31^ or between sera of prostate cancer and BPH patients^9,32,33^. They reported that the amounts of PSA in Con A (−) fraction were significantly higher in prostate cancer samples than in BPH samples. These studies only measured the amounts of PSA included in Con A (−) and (+) fractions, not the structure of the molecule, therefore it was not clear whether Con A (−) fraction of PSA have multi-antennary *N*-glycans or is non-*N*-glycosylated. From our research, the detailed structures of PSA glycoforms in cancer cells, especially included in the Con A (−) fraction, has been elucidated for the first time.

In the Con A (−) fraction, we found PSA with both high- and low- molecular weights in common among cancer origins (Fig. 3). Series of MALDI-MS analysis indicated that highly branched complex type glycans exist in the Con A (−) fraction and α1,2- and/or α1,3(4)-fucosylation occur on PSA from LNCaP (Fig. 5). It is also shown that the low molecular PSA from LNCaP is either with shortened or without *N*-glycans. MALDI-MS analysis detects no peptides with one *N*-acetylglucosamine (±fucose) on the Asn, suggesting that the low molecular PSA is not produced by endo-β-*N*-acetylglucosaminidase.

Since the occurrence of PNGase activity in several mammalian cultured cells was reported^34^, we investigated whether the low molecular PSA was deglycosylated by PNGase type enzyme. The LC-MS analysis showed that the majority of the *N-*glycosylation potential site on PSA remained Asn in Con A (−) fraction from LNCaP (Fig. 7), suggesting the consequences of glycan transfer failure in biosynthetic pathway. The truncated mucin-type *O*-glycans were found in many cancers^35^; however, inadequate research has been performed on the hypoglycosylation of *N*-glycans till date. As one of the reasons, it is considered that non-glycosylated peptides are often separated in different fractions by hydrophilic chromatography for enrichment of glycopeptides prior to mass spectrometry and may fail to be detected.

We examined whether a non-*N*-glycosylated form exist in other glycoproteins which is secreted from prostate cancer cells. Zinc-alpha-2-glycoprotein (ZAG) is the glycoprotein that contains four putative glycosylation sites and is produced in many cell types including prostate epithelial cells^36^. In conditioned medium of LNCaP cell, only 3% of ZAG from LNCaP passed through a Con A column, while 22% of PSA in same fraction (Supplementary Fig. S3a). Moreover, the molecular form that corresponding to the non-*N*-glycosylated form of ZAG was not observed in Con A (−) fraction (Supplementary Fig. S3b). Non- *N*-glycosylation seems to be not always common to secreted glycoproteins in LNCaP cell.

An elevation of branching of *N*-linked glycans is often observed in glycoproteins produced by cancer cells^37^. In this study, we found that multi-antennary glycans were expressed in CTOS as well as 22Rv1 and LNCaP cells. We previously identified PSA with tri-antennary glycan in prostate cancer serum by MALDI-MS^38^. Haga *et al*.^39^ analysed glycan structures of PSA using LC-MS/MS and their data showed that multi-antennary glycans are present in greater amounts in the sera of prostate cancer patients compared with BPH patients.

A frequent overexpression sialyl Le^x/a^ (SLe^x/a^) antigen on cancer cells has been shown^40^. We also proved the existence of fucose in the outer chains of PSA from cancer cells (Figs. 5b,c and 8), and significant expression of α1,3 FTs (Supplementary Fig. S1) in CTOS and 22Rv1, which regulate the synthesis of Le^x^ and SLe^x^ in PC-3 prostate cancer cell^22^. Since up-regulation of SLe^x^ was observed in metastatic prostate cancer^41^, the expression of SLe^x^ on PSA may become a candidate for prognostic marker. Recently, the elevated fucosylation in the outer chains of PSA glycans was found using lectin-immunoassay^42^.

Many studies have been done which focus glycans as biomarkers in prostate cancer^3^, especially *N*-glycan alterations of PSA^9–12,39,43,44^. Previous studies have revealed that not all patients can be detected by measuring the increase focusing only on a specific glycan structure such as α2,3-sialyl or LacdiNAc residues^9–12,43,44^. Assessing cancer-specific changes in combination with other target may improve accuracy. Our study has newly discovered that PSA with particular multi-antennary *N*-glycan and non-*N*-glycosylated PSA, which are rarely present in normal seminal plasma. In particular, the discovery of a marker from a new viewpoint of the presence or absence of *N*-glycan is expected to increase options. It is reported that the loss of *N*-glycosylation affects the malignancy of cancer^45,46^. Therefore, it is important to determine the rate of *N*-glycosylation besides detecting the particular glycan structure.

In this study, by analysing the *N*-glycans of PSA from CTOS and other cancer cells, we present the new candidates for cancer-related forms of PSA. Development of probes that recognise the novel PSA forms will pave the way for a sensitive diagnosis. Since the blood stability of these PSA forms are unknown, it is necessary to verify whether these forms are actually elevated in the sera of cancer patients in our next study.

## Methods

### Materials

Seminal PSA was purchased from Calbiochem (San Diego, CA). The WHO International Standard PSA Free (NIBSC code 96/668) was obtained from National Institute for Biological Standards and Control (Herts, UK). Seminal PSAs were purified from seminal plasma of healthy men and were used as normal control. Synthetic peptides IRNKS and IRDKS were obtained from BEX Co. Ltd. (Tokyo). Bovine serum albumin (BSA) and phenylmethylsulfonyl fluoride (PMSF) were purchased from Merck (Darmstadt, Germany) and Halt^TM^ protease inhibitor was obtained from Thermo Fisher Scientific (Rockford, IL).

### Preparation of galectin-1 column

Recombinant His-tagged galectin-1 was prepared as described previously^47^. The concentration of the purified galectin-1 was determined by a Bio-Rad Protein Assay dye reagent (Bio-Rad, Hercules, CA) using BSA as standard. The purified galectin-1 was immobilised to CNBr-activated Sepharose 4B (GE healthcare, Pittsburgh, PA) according to the manufacturer’s instructions.

### Cancer cell lines

Human prostate cancer cell line LNCaP.FGC (RCB2144) was purchased from RIKEN BioResource Center (Tsukuba, Japan) and 22Rv1 was from American Type Culture Collection (Manassas, VA). Cells were cultured in RPMI 1640 medium with 10% fetal bovine serum (Thermo Fisher Scientific) and 1% penicillin/streptomycin (Invitrogen, Carlsbad, CA, USA). For large scale preparation of PSA, cells were cultured with serum free RPMI 1640 medium supplemented with dihydrotestosterone (DHT, 125 nM, FUJIFILM Wako Pure Chemical) for 4 days. LNCaP cells that had passage numbers between 20 and 40 were used in this study. A conditioned medium was collected and was centrifuged at 3,000 rpm for 5 min to remove flow cells in medium, then was concentrated with Amicon Ultra 15 mL 10 K centrifugal filter (Merck) and stored at −20 °C until use.

### Preparation of CTOS from tumour specimens

The patient-derived xenografts (PDXs) were generated from the tumour tissue with the patients’ informed consent and approval of ethics committees by both Osaka University and the National Institutes of Biomedical Innovation, Health, and Nutrition. The study was also approved by the institutional ethics committees at Osaka International Cancer Institute. The PDX line, #415, was established from a metastatic tumour in the testis of a PSA-positive 59-year-old patient, initially diagnosed as prostate cancer at a clinical stage T4N1M0 (Gleason score 5 + 5). #415 grew rapidly in LPS non-responder Super SCID mouse C3H/HeJ/NOs-scid, and PSA in the blood serum of the host mice increased up to 1000 ng/mL along with the increase of tumour size after transplantation. Animal studies were performed in accordance with the guidelines of the Institutional Animal Study Committee of the National Institutes of Biomedical Innovation, Health, and Nutrition. CTOSs were prepared from the xenograft tumours as previously described^48^. Briefly, tumours were mechanically minced and digested in DMEM/Ham’s F12 medium (FUJIFILM Wako Pure Chemical, Osaka) with Liberase DH (Roche, Basel). Fractions were recovered between 40–100 μm using mesh filters (BD Falcon, Franklin Lakes) and cultured overnight at 37 °C under 5% CO2, in STEMPRO hESC SFM (Invitrogen, Carlsbad, CA). Then, we picked up 5000 CTOSs and plated 1000 CTOSs/well × 5 wells in 24 well non-coated dishes and cultured in StemPro medium (1 ml/well) with 125 nM DHT. At 72 hours after plating, we collected the cell suspension, centrifuged at 1,500 rpm for 5 min at 4 °C, collected supernatant, and kept in −20 °C until further analysis.

### Enzyme-linked immunosorbent assay for PSA

Anti-PSA monoclonal antibody (#56, Mikuri Immunology Laboratory, Osaka) was diluted to a final concentration of 5 μg/ml in PBS and was transferred to a 96 well microtiter plate (Nunc-Immuno Plate Maxisorp Surface, Thermo Fisher Scientific), and the plate was left overnight at 4 °C. Then, unbound sites were blocked with fivefold diluted Blocking Reagent N102 (Nichiyu, Tokyo). After washing with PBS-T (0.05% Tween 20 in PBS), WHO International Standard PSA Free and samples were diluted in blocking solution and were added to each well of microtiter plate and the plate was incubated for 2 h at room temperature. After washing with PBS-T, HRP-conjugated anti-PSA antibody (Dako, Agilent Technologies, CA) was incubated for 1 h at room temperature. The plate was washed with PBS-T and incubated with eBioscience™ tetramethylbenzidine solution (Thermo Fisher Scientific) and the released chromogen was measured with a photo spectrometer (CORONA Electric SH-1300Lab).

### Lectin affinity chromatography

A concentrated culture medium in 250 μL of equilibration buffer was applied to lectin column (1 ml) and allowed to stand at 4 °C for 20 min. Unbound (−) fraction of lectin column was obtained by adding 4.75 ml of equilibration buffer and bound (+) fraction was obtained by adding 5 ml of elution buffer. Elution condition of each lectin affinity chromatography is described as follows. A Con A-Sepharose 4B (15 mg/ml, GE healthcare) was equilibrated with TBS + buffer (0.1 mg/ml BSA, 0.1 mM PMSF, 20 mM Tris-HCl, pH 7.4 containing 0.15 M NaCl, 1 mM CaCl_2_, 1 mM MnCl_2_) and eluted with 0.3 M α-D-methylglucoside (α-MG) in TBS + buffer. A DSA-Sepharose (3 mg/ml, J-Oil Mills, Tokyo) was equilibrated with PBS + buffer (0.1 mg/ml BSA, 0.1 mM PMSF, PBS), and eluted with 0.1 N acetic acid containing 0.1 mg/ml BSA and neutralised with 1 M Tris-HCl pH 8.0. WFA-agarose (4.6 mg/mL, J-Oil Mills) and galectin-1-Sepharose (1.5 mg/ml) were equilibrated with PBS + buffer and eluted with 0.4 M lactose/PBS + buffer and 0.2 M lactose/PBS + buffer, respectively.

Concentrations of PSA in each fractions were determined by ELISA as described above. The relative abundance of PSA in each (−) and (+) fractions is presented as percent with the sum of PSA in (−) and (+) fractions being 100 percent. We performed experiments several times using the condition medium from cells of the different passages. A representative chromatogram for each sample was shown in figure.

### Glycosidase digestion

To remove sialic acids, PSA was equibrated with reaction buffer (0.15 M sodium acetate buffer, pH 5.0, Halt^TM^ protease inhibitor) incubated with 125 mU of *Arthrobacter ureafaciens* sialidase (Nacalai Tesque, Kyoto, Japan) at 37 °C for 18 h. After the reaction, samples were neutralised with sodium hydroxide. For de-*N*-glycosylation, samples are mixed with rapid PNGase F (P0710S, New England Biolabs Japan) in reaction buffer and incubated at 50 °C for 10 min according to manufacture’s instruction. To remove fucose from glycopeptides, thermolysin-digested glycopeptides were treated with 40 mU of α1,2-fucosidase (Takara Bio, Otsu, Japan) in 90 mM sodium phosphate buffer pH 8.5 or with 4 U of α1,3/4-fucosidase (P0769, New England Biolabs Japan) in reaction buffer (50 mM acetate buffer pH 5.5 containing 5 mM Ca^2+^ and 0.1 mg/ml of BSA) at 37 °C for 16 h, respectively.

### Immunoblotting

PSA molecules were separated by SDS-PAGE using 12% gel and were transferred onto nitrocellulose membrane (pore size: 0.2 μm, Bio-Rad). Blots were blocked with 5% skim milk in TBS-T (20 mM Tris-HCl, 150 mM NaCl pH 7.5 containing 0.1% Tween 20). The membrane was incubated with anti-PSA polyclonal antibody (Dako) at 4 **°**C overnight. After being washed with TBS-T, the blots were incubated with HRP-conjugated anti-rabbit IgG and the bound conjugates were visualised with SuperSignal West Dura Extended Duration Substrate (Thermo Fisher Scientific) and imaged using a Bio-rad ChemiDoc XRS™. Determining of molecular weight was performed with Image Lab™ Software (Bio-Rad).

### Immunoprecipitation

Anti-PSA monoclonal antibody was immobilised to NHS-Mag Sepharose (GE healthcare) according to the manufacturer’s instructions. The antibody-coupled magnetic beads (10 μg/μL) were incubated with PSA samples for 16 h at 4 °C with gentle rotating. The beads were washed twice with PBS-T and once with PBS. The washed beads were eluted with 50% formic acid/30% acetonitrile and elutes were evaporated and stored at −20 °C until use. Elution was also performed by boiling with SDS sample buffer for gel electrophoresis.

### Protease digestion and MALDI-MS

PSA was purified by immunoprecipitation from the concentrated conditioned medium of LNCaP which had passage numbers between 20 and 40. PSA was dissolved in 25 mM NH_4_HCO_3_ solution (50 μL) with 10 μg of thermolysin (Calbiochem) and was allowed to stand at 60 °C for 18 hours. The evaporated samples were dissolved in 100 μL of aqueous solution containing 80% acetonitrile and applied to the pre-equilibrated GL-Tip-Amide (GL Sciences Inc., Tokyo, Japan), and were washed with 80% acetonitrile. Next, the adsorbed glycopeptides were eluted with 80% acetonitrile- and 50% acetonitrile-water solution containing 0.1% trifluoroacetic acid, respectively. The eluted fractions were combined and were dried using a centrifugal evaporator and were dissolved in 10 μL of water. Digestion of PSA was also performed with 10 μL of Asp-N (10 ng/μL, Wako Pure Chemical Industries) at 37 °C for 18 hours and peptides were purified using C-18 tip (Thermo Fisher Scientific).

An aliquot of the dissolved sample solution (1.0 μL) was mixed with 0.5 μL of 1% methylene-diphosphonic acid on the target plate to reduce salt-induced signal suppression^49^. After drying, the samples were mixed with 0.8 μL of 2,5-dihydroxybenzoic acid solution (5 mg/ml, Shimadzu Biotech, Kyoto). We acquired mass spectra using a MALDI-TOF MS (AXIMA TOF^2^, Shimadzu Biotech) in linear mode or a MALDI QIT-TOF MS (AXIMA Resonance, Shimadzu Biotech) in positive and negative ion modes.

### LC-MS analysis of non-glycosylated PSA peptides

LC-MS experiments were performed using an Ultimate 3000 HPLC system fitted to a Velos Pro Ion Trap LC-MS^n^ system (Thermo Fisher Scientific). PSA from LNCaP was digested with thermolysin and peptides were purified as described above. For analysis of non-glycosylated peptides, samples were dissolved in 100 μL water in a 250 μL auto sampler vial. The LC-MS/MS analysis was performed using a C18 column, Xselect HSS T3 XP (2.1 × 100 mm, 2.5 µm, Waters). After equilibration with 20 mM ammonium formate, isocratic elution was performed with 0.1% formic acid, 100 µL/min and a 5 min linear gradient with 0–100% acetonitrile and a 5 min isocratic elution with 100% acetonitrile were performed to elute residual peptides. The temperature of the heated capillary was set at 150 °C and the ion spray voltage at 3.5 kV.

## Supplementary information

Supplementary information.
