# Supplementary material for: Study of glycosylation of prostate-specific antigen secreted by cancer tissue-originated spheroids reveals new candidates for prostate cancer detection
Source: Sci Rep. 2020 Feb 17;10:2708. doi: 10.1038/s41598-020-59622-y (PMC7026178; doi:10.1038/s41598-020-59622-y)

**Study of glycosylation of prostate-specific antigen secreted by cancer tissue-originated spheroids reveals new candidates for prostate cancer detection**

Hiroko Ideo^1^, Jumpei Kondo^2, 3^, Taisei Nomura^4^, Norio Nonomura^5^, Masahiro Inoue^2, 3^, and Junko Amano^1,*^

1 Laboratory of glycobiology, The Noguchi Institute, Tokyo 173-0033, Japan

^*^Corresponding author [amano@noguchi.or.jp](mailto:amano@noguchi.or.jp)

2 Department of Clinical Bio-resource Research and Development, Graduate School of Medicine, Kyoto University, Kyoto 606-8501, Japan

3 Department of Biochemistry, Osaka International Cancer Institute, Osaka 541-8567, Japan

4 Animal Models of Human Diseases, National Institutes of Biomedical Innovation, Health and Nutrition, Osaka 567-0085, Japan

5 Department of Urology, Osaka University Graduate School of Medicine, Osaka 565-0871, Japan

**Supplementary Information**

**Supplementary methods**

### Real-time PCR analysis of fucosyltransferase gene expression

Total RNA from prostate cancer cells were prepared with the RNeasy^®^ Mini Kit (Qiagen, Valencia, CA) according to the manufacturer's instructions. First-strand cDNA was synthesised from total RNA using the SuperScript VILO cDNA Synthesis Kit (Thermo Fisher Scientific). Quantitation of human glycosyltransferase transcript by real-time PCR was performed on the CFX connect real-time PCR detection system (Bio-Rad) with SsoAdvanced^™^ universal SYBR^®^ Green Supermix (Bio-Rad). The amount of each glycosyltransferase transcript was normalised to the amount of GAPDH transcript in the same cDNA sample. Relative fold differences in transcript expression were approximated using the Comparative C_T_ method. Forward and reverse primers for real-time PCR were obtained from Fasmac Co. Ltd. (Kanagawa, Japan) with the following sequences: GAPDH, 5′-GAAGGTGAAGGTCGGAGT-3′, 5′-GAG ATG GTGATGGGATTTC-3′; FT3, 5′-GCCGACCGCAAGGTGTAC-3′, 5′-TGACTTAGGGTTGGACATGATATCC-3′; FT6, 5′-CAAAGCCACATCGCATTGAA-3′, 5′-ATCCCCGTTGCAGAACCA-3′; FT7, 5′-TCCGCGTGCGACTGTTC-3′, 5′-GTGTGGGTAGCGGTCACAGA-3′.

**Preparation of recombinant PSA in E. coli**

We amplified fragment which correspond to the pro-from of PSA adding ATG at *N*-terminal using cDNA from LNCaP by PCR. After the sequence of the amplified fragment was confirmed, the fragment was inserted into the plasmid pET21a (Novagene, Darmstadt, Germany) using In-Fusion HD cloning kit (Takara Bio). The pET21[PSA] was transformed with E. coli strain SHuffle T7 Express (New England Biolabs Japan). PSA-expressing E. coli was cultured at 18°C for 20 h after the addition of IPTG to the final concentration of 0.4 mM. The centrifugated pellet was resuspended and sonicated in PBS containing PMSF and then was centrifugated at 11,000 × g for 10 min. The recombinant PSA was purified from the supernatant by immunoprecipitation as described in “Methods”.

**Preparation of PNGF treated PSA from LNCaP cells**

PSA was purified by immunoprecipitation from the Con A (+) fraction of LNCaP and was treated with PNGF as described in “Methods”.

**Analysis of zinc-alpha-2-glycoprotein (ZAG) in Con A (−) and (+) fractions**

The conditioned medium from LNCaP cancer cells were applied to a Con A lectin column and was fractionated as described in “Methods”. The amount of ZAG in Con A (−) and (+) fractions were measured by Alpha-2 Glycoprotein/ZAG/A2GP1 Human ELISA Kit (Thermo Fisher Scientific) according to the manufacturer’s instructions. The relative abundance of PSA and ZAG in each (−) and (+) fractions is presented as percent with the sum of proteins in (−) and (+) fractions being 100 percent.

ZAG molecules in Con A (−) and (+) fractions were analysed in the same way as PSA by western blotting using rabbit anti-ZAG antibody (GeneTEX, Hsinchu City, Taiwan) as the first antibody.

**
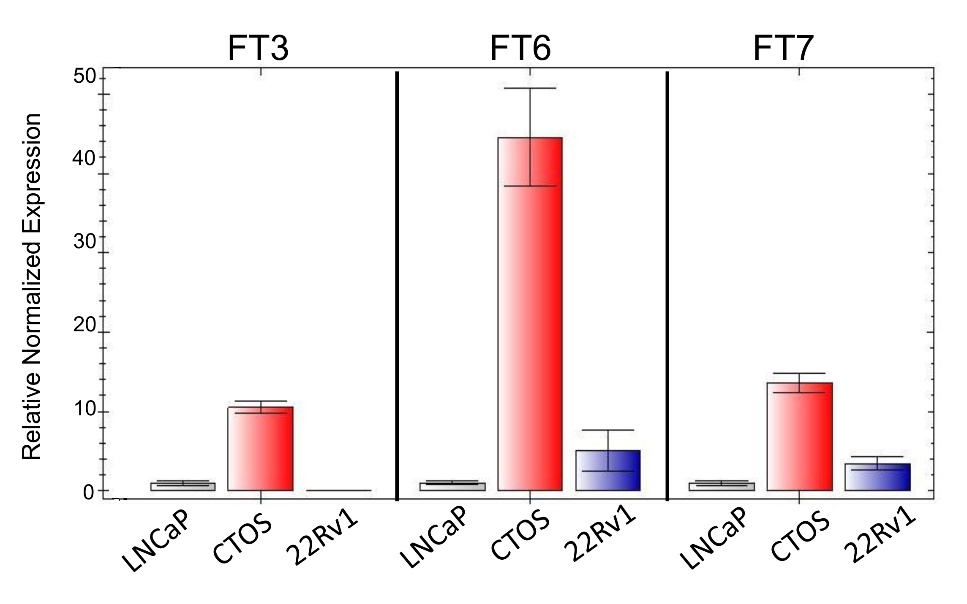
**

**Supplementary Fig. S1**

**Elevation of** α**1,3 fucosyltransferases (FT) 3, 6 and 7 in CTOS and 22Rv1 cells.**

A real-time PCR analysis of FTs was performed on cDNA isolated from cancer cells. After normalising with the *GAPDH* gene, results are expressed as mean fold change in each fucosyltransferase relative to expression level in LNCaP cells in triplicate. The expression level in LNCaP is set to 1. Bars represent mean ± SEM (standard error of the mean).


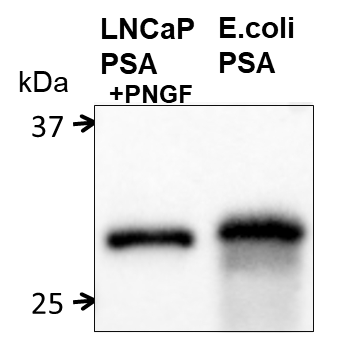


**Supplementary Fig. S2**

**Western blot analysis of the PNGF treated PSA from LNCaP and the recombinant PSA.**

(left): PNGF treated PSA from LNCaP, (right): the recombinant PSA prepared in *E.coli.*


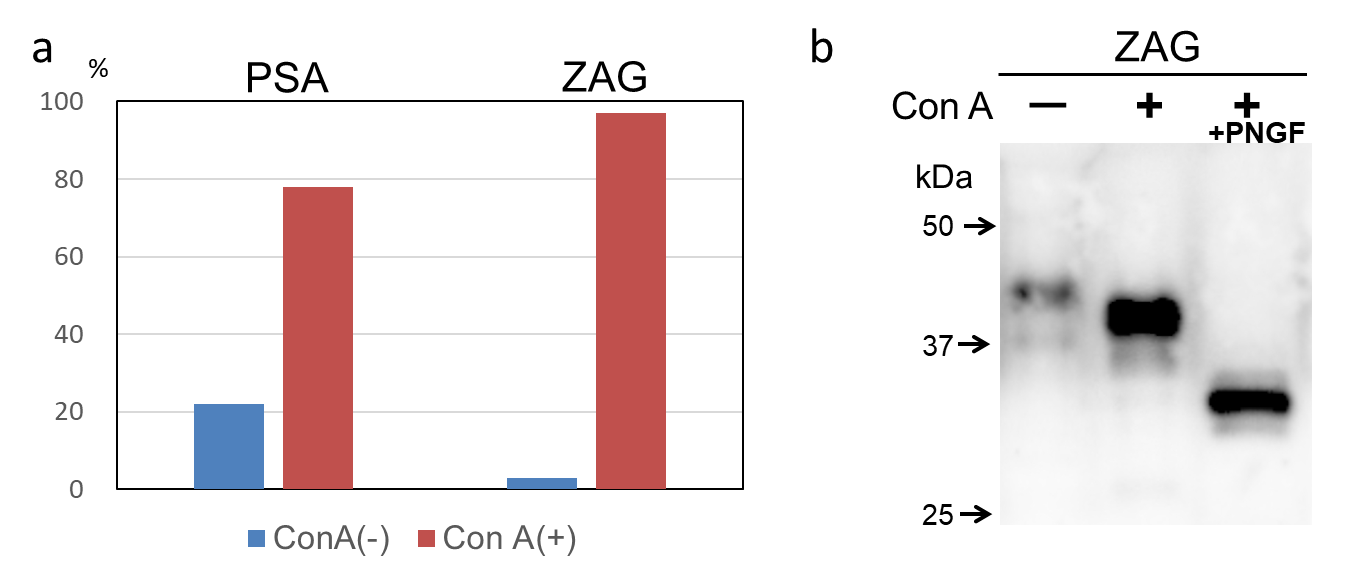


**Supplementary Fig. S3**

**Analysis of ZAG in Con A (−) and (+) fractions of LNCaP cell**

(a) relative abundance of PSA and ZAG in Con A (−) and **(+)** fractions of LNCaP cell.

Only 3 % of ZAG from LNCaP passed through a Con A column, while 22 % of PSA in same fraction.

(b) molecular forms of ZAG in Con A (−) and **(+)** fractions of LNCaP cell.

(left): Con A (−) fraction, (middle): Con A (+) fraction, (right): PNGF treated Con A (+) fraction.

The sample amount of Con A (−) fraction was used three times as much as that of Con A (+) fraction. The molecular form that corresponding to the deglycosylated form of ZAG was not observed in Con A (−) fraction.

**Supplementary Fig. S4** Uncropped image for Fig. 3a-d, Fig4d-f and Supplementary Fig. S2 and Fig. S3b.

**Related to Fig. 3a and 3b**


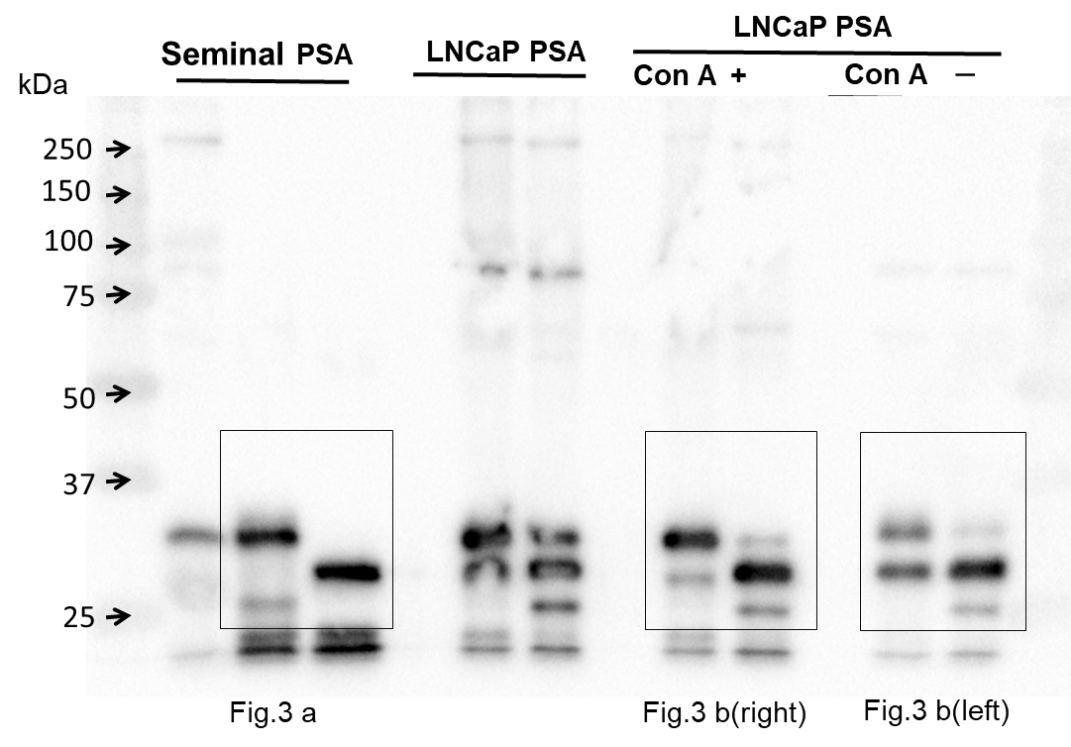


**Related to Fig. 3c 　　　　　　　　　　Related to Fig. 3d**

**
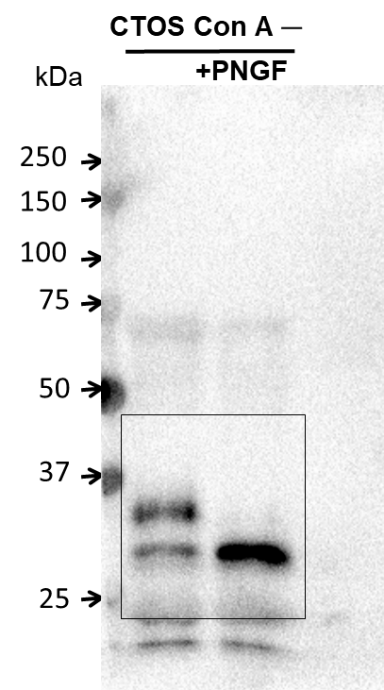
**
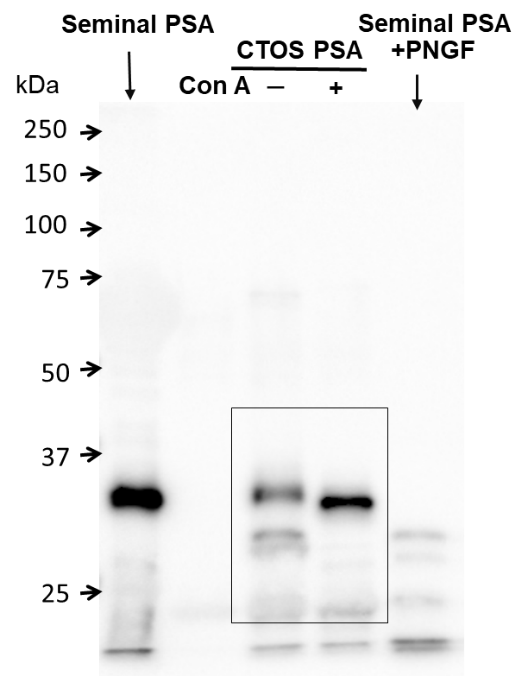


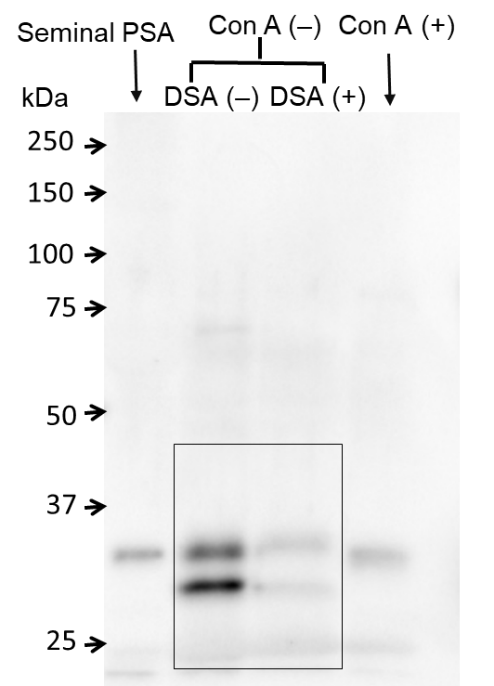
**Related to Fig. 4d Related to Fig. 4e Related to Fig. 4f**


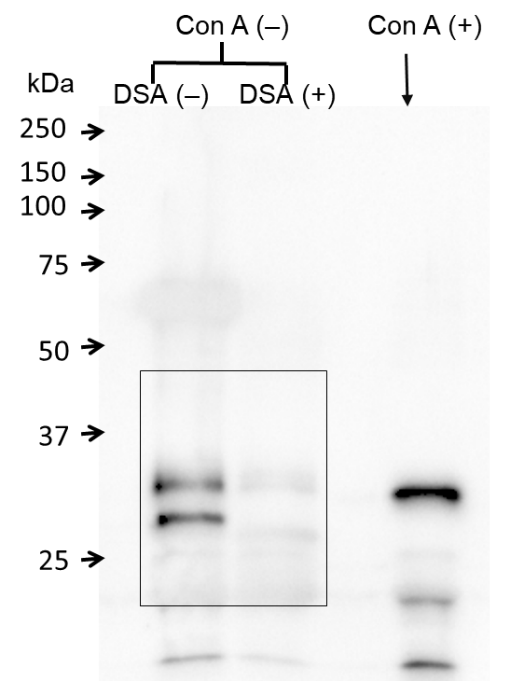

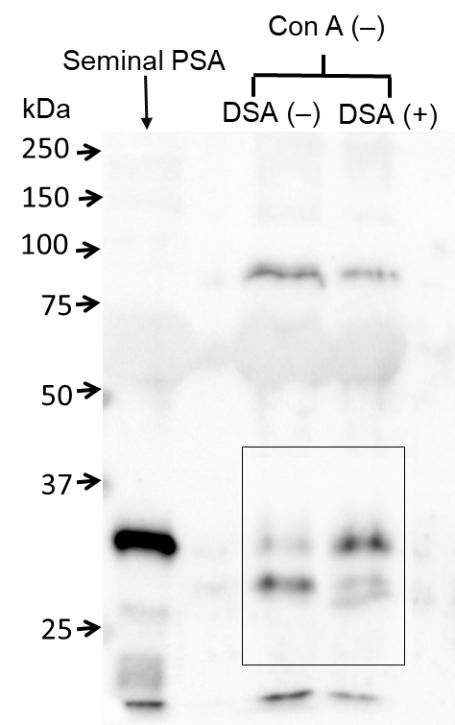


**Related to Supplementary Fig. S2 Related to Supplementary Fig. S3b**


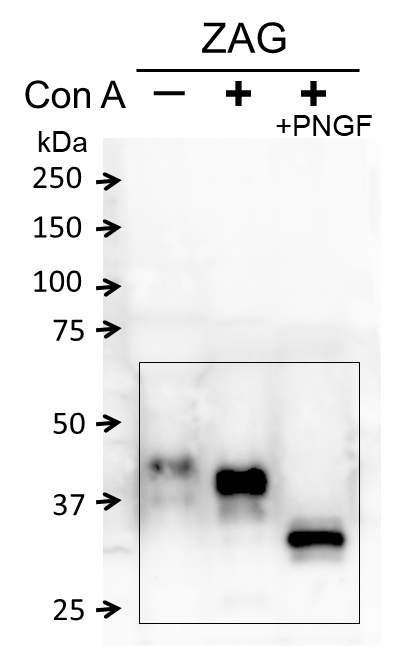

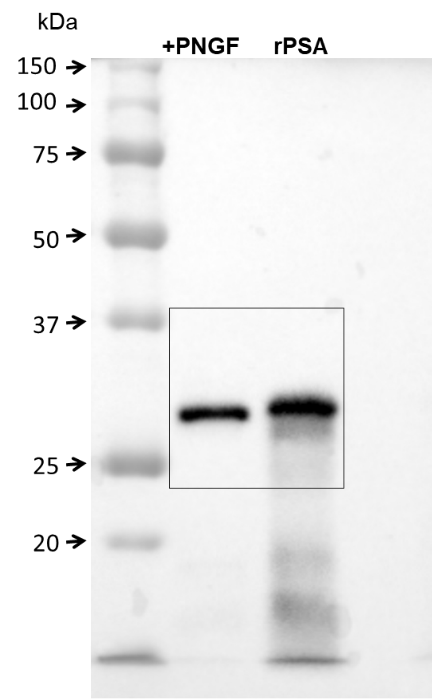

Supplement: Supplementary file 1 — Supplementary information. [file 41598_2020_59622_MOESM1_ESM.docx]
